# Coffee consumption modifies risk of estrogen-receptor negative breast cancer

**DOI:** 10.1186/bcr2879

**Published:** 2011-05-14

**Authors:** Jingmei Li, Petra Seibold, Jenny Chang-Claude, Dieter Flesch-Janys, Jianjun Liu, Kamila Czene, Keith Humphreys, Per Hall

**Affiliations:** 1 Department of Medical Epidemiology and Biostatistics, Karolinska Institutet, Box 281, Stockholm 17177, Sweden; 2 Human Genetics, Genome Institute of Singapore, 60 Biopolis St, Singapore 138672, Singapore; 3 Division of Cancer Epidemiology, German Cancer Research Center (DKFZ), Im Neuenheimer Feld 581 (TP4), Heidelberg 69120, Germany; 4 Department of Medical Biometrics and Epidemiology, University Medical Center Hamburg-Eppendorf, Martinistr. 52, Hamburg 20246, Germany

## Abstract

**Introduction:**

Breast cancer is a complex disease and may be sub-divided into hormone-responsive (estrogen receptor (ER) positive) and non-hormone-responsive subtypes (ER-negative). Some evidence suggests that heterogeneity exists in the associations between coffee consumption and breast cancer risk, according to different estrogen receptor subtypes. We assessed the association between coffee consumption and postmenopausal breast cancer risk in a large population-based study (2,818 cases and 3,111 controls), overall, and stratified by ER tumour subtypes.

**Methods:**

Odds ratios (OR) and corresponding 95% confidence intervals (CI) were estimated using the multivariate logistic regression models fitted to examine breast cancer risk in a stratified case-control analysis. Heterogeneity among ER subtypes was evaluated in a case-only analysis, by fitting binary logistic regression models, treating ER status as a dependent variable, with coffee consumption included as a covariate.

**Results:**

In the Swedish study, coffee consumption was associated with a modest decrease in overall breast cancer risk in the age-adjusted model (OR_> 5 cups/day _compared to OR_≤ 1 cup/day_: 0.80, 95% CI: 0.64, 0.99, *P *trend = 0.028). In the stratified case-control analyses, a significant reduction in the risk of ER-negative breast cancer was observed in heavy coffee drinkers (OR_> 5 cups/day _compared to OR_≤ 1 cup/day _: 0.43, 95% CI: 0.25, 0.72, *P *trend = 0.0003) in a multivariate-adjusted model. The breast cancer risk reduction associated with higher coffee consumption was significantly higher for ER-negative compared to ER-positive tumours (*P *heterogeneity (age-adjusted) = 0.004).

**Conclusions:**

A high daily intake of coffee was found to be associated with a statistically significant decrease in ER-negative breast cancer among postmenopausal women.

## Introduction

Coffee is one of the most popular beverages in the world. The latest coffee trade statistics estimated that world coffee production amounted to 7.4 billion kg in 2009/2010 [1]. In Sweden, where coffee consumption is among the highest in the world, the average coffee consumption in 2008 was 8.2 kg per person [1,2], with a median of three cups per person per day.

Coffee is interesting in the light of breast cancer etiology because of its complex make-up of chemicals, several of which have been shown in experimental studies to have cancer risk altering potential through meaningful biological mechanisms. The scientific community, however, stands divided over toxicity of the beverage. It has been demonstrated in experimental and clinical studies that coffee, being a complex mixture of caffeine and polyphenols [3-7], can play a dual role as both a carcinogen, in which it inhibits cellular repair of DNA or enhances cell proliferation [8-11], and a chemo-preventive agent with anti-oxidative and weakly estrogenic properties [12,13]. The bulk of previous studies suggest that high coffee consumption is associated with a modest reduction of breast cancer risk [14,15], although a meta-meta-analysis of over 500 papers relating the consumption of coffee to cancer of various sites by Arab [16] reported a null association with breast cancer risk.

Breast cancer is a complex disease and may be sub-divided into hormone-responsive (estrogen receptor (ER) positive) and non-hormone-responsive subtypes (ER-negative). Coffee itself might contain compounds that differentially affect breast cancer of different ER subtypes. We, thus, hypothesize that heterogeneity exists between coffee consumption and breast cancer risks for ER-positive and ER-negative breast cancers.

In this study, we examine the association between coffee consumption and postmenopausal breast cancer risk in a large population-based study (2,818 cases and 3,111 controls), overall and stratified by hormone receptor status. As the possibility that the weak relationship between high levels of coffee consumption and the occurrence of breast cancer is due to confounding by related dietary or lifestyle factors is tenable [17], we also adjusted for these factors in our final multivariate models.

## Materials and methods

### Subjects

Subjects were drawn from a population-based case-control study, which has been described in detail previously [18]. The parent study consisted of women aged 50 to 74 years, born in Sweden and resident there between 1 October 1993 and 31 March 1995. An attempt was made to contact all incident cases of invasive breast cancer in this population. Cases were identified through six Swedish regional cancer registries, and written consent to be approached with a mailed questionnaire was requested from the women through their physicians. The participation rate, amongst 3,979 eligible cases detected, was 84%. Non-participation was attributed to either refusal by the physician (4%) or the patient (12%). Controls were frequency-matched to the cases by age. Of 4,188 controls who were randomly selected from a continuously updated Swedish register, 3,454 (82%) gave consent to participate in the study. Exclusions were made for women who were pre-menopausal (198 cases, 152 controls), or with unknown menopausal status (217 cases, 100 controls), or with a previous diagnosis of cancer (other than non-melanoma skin cancer or cancer *in situ *of the cervix) (112 cases, 91 controls). The final study group consisted of 2,818 cases and 3,111 controls. The ethical review board at the Karolinska Institute and the six ethical review boards in other regions of Sweden approved the study.

For the validation analysis, subjects were drawn from the population-based case-control MARIE (Mamma Carcinoma Risk factor Investigation) study which was carried out from August 2002 to September 2005 in two study regions in Germany (the Hamburg and Rhein-Neckar-Karlsruhe regions). Details of the study design can be found in Flesch-Janys *et al. *[19]. Briefly, the MARIE study included 3,464 postmenopausal and histologically confirmed incident breast cancer cases aged 50 to 74 at diagnosis with primary invasive or *in situ *tumours (International Classification of Diseases (ICD) 10: C50 and D05) and 6,657 controls, frequency matched by year of birth and study region. Two controls per case were randomly selected from the lists of residents provided by the population registries. For the present analysis, *in situ *cases were excluded. The study was approved by the ethics committees of the University of Heidelberg and the University of Hamburg. All study participants gave written informed consent.

### Data collection

Data were obtained by means of an extensive mailed questionnaire requesting detailed information on established and possible breast cancer risk factors, including reproductive and menstrual history, family history of breast cancer, hormone replacement therapy (HRT) and anthropometric measures, such as body mass index (BMI). Information on lifestyle such as smoking (> 1 year or > 100 cigarettes), alcohol intake (g/day) and physical activity (none, less than one hour per week, one to two hours per week or more than two hours per week) was also collected from the questionnaire. Highest education level attained was available as a categorical variable (elementary school, junior secondary school, high school or university). Data on the consumption of coffee one year prior to interview, specified in cups per week, where a cup was equivalent to 1.5 dl, were also collected. Age at menopause was defined as the age of the last menstrual period or age at bilateral oophorectomy, if one year or more prior to data collection. The women were considered pre-menopausal if menopause occurred less than one year before data collection. Women with hysterectomy, menses due to HRT or missing information were considered post-menopausal if they had reached the 90^th ^percentile of the age of natural menopause (54 years in current smokers and 55 years in non-smokers, regardless of case/control status), or otherwise as unknown. Subjects classified as post-menopausal in this manner (280 cases and 303 controls) were assigned an age at menopause according to their case/control and current smoking status corresponding to the mean age at natural menopause in the respective groups.

Information regarding the retrieval of hormone receptor status from the medical records of all participants from surgical and oncological units throughout Sweden has been presented in detail elsewhere [20,21]. Although ER and PR content of breast tumours were routinely measured in Sweden at the time of the study, this was often not performed on tumours ≤ 1 cm in size due to lack of tumour tissue. Quantitative receptor content was thus only available for 65.4% (1,835 women) of the tumours for both ER and PR.

For the validation study (MARIE), information on potential risk factors for breast cancer was obtained in face-to-face interviews using a standardized questionnaire. Nutritional data were collected using a food frequency questionnaire with 176 food items regarding dietary habits in the year prior to date of diagnosis for cases and date of food frequency questionnaire completion for controls. The consumption of caffeine-containing coffee was calculated in cups per day based on the information on both portion size (non-consumer, 0.5, 1, 2, 3 cups) and frequency (non-consumer, once per month or less, two to three times per month, once per week, two to three times per week, four to six times per week, once per day, twice per day, three to four times per day, five times per day or more). The analysis was limited to women who answered both questions on portion size and frequency of caffeine containing coffee consumption. The final study group comprised 5,395 controls and 2,651 cases. Information on tumour characteristics, such as ER and PR status, was obtained from medical records.

### Statistical analysis

The variable for coffee consumption was categorized as follows: one cup or less per day; more than one to three cups/day; more than three to five cups/day; five or more cups/day. These categories were based on the distribution within the control group. Since very few women abstained from coffee, we combined abstainers and low consumers (one cup per day) into a single category. Women who consumed one cup or less of coffee per day served as the reference group for all regression analyses.

Unconditional logistic regression models, adjusting for the matching factor, age at enrolment in years (continuous), were applied to evaluate if established or possible breast cancer risk factors had (including coffee consumption) significantly different distributions/means (using the Wald test) between breast cancer cases and controls in this study.

The relationships between coffee consumption and other breast cancer risk factors were explored in the control population by treating coffee consumption as a covariate and using linear regression analysis for continuous risk factor variables (age at menarche (years), age at menopause (years), BMI (kg/m^2) ^and alcohol consumption (g/day)), logistic regression analysis, for binary risk factor variables (HRT, family history of breast cancer and smoking) or proportional odds logistic regression, for categorical risk factor variables (parity/age at first birth (nulliparous; parous and age at first birth < 25 yr; parous and age at first birth ≥ 25 yr and < 30 yr; parous and age at first birth ≥ 30 yr), highest education level (elementary school, junior secondary school, high school and university), and recent physical activity (one year before enrolment; none, less than one hour per week, one to two hours per week, more than two hours per week)). The Wald test was used to determine the statistical significance of an overall linear trend for the association between coffee consumption, treated as a semi-continuous variable, and the breast cancer risk factor in the models fitted.

For models for breast cancer risk, covariates were considered to be potential confounders if they were found to be associated with both coffee consumption and breast cancer risk, and caused a shift of > 10% in estimates for any coffee category when added to the model. ORs and corresponding 95% CI were estimated for the multivariate logistic regression models fitted to examine breast cancer risk, overall, and stratified by ER and PR tumour subtypes. Three models were fitted for each outcome: adjusted for the matching factor (age at enrolment only), adjusted for age at enrolment, HRT, smoking and education, and adjusted for age at enrolment, HRT, smoking, education and daily alcohol consumption. The Wald test was used to determine the statistical significance of an overall linear trend for the association between coffee consumption, treated as a semi-continuous variable, and the breast cancer risk.

Since ER and PR status are strongly correlated (logistic regression *P*-value for association < 2.0 × 10^-16^), we assessed the extent to which coffee consumption drives each of the two tumour characteristics, by fitting multinomial regression models for five outcomes (controls, ER-negative and PR-negative, ER-negative and PR-positive, ER-positive and PR-negative, ER-positive and PR-positive). We compared a model without parameter restrictions to models with parameters restricted such that coffee consumption was only allowed to be associated with one tumour characteristic at a time. Likelihood ratio tests, with two degrees of freedom, were used to test the null hypothesis that associations between coffee consumption and PR status was due only to an association with ER or PR status.

Associations between coffee consumption and hormone receptor status were evaluated in a case-only analysis, by fitting binary logistic regression models (for ER and PR status), treating ER or PR status as dependent variables, with coffee consumption included as a covariate. ORs and corresponding 95% CI were estimated for each coffee consumption category. *P*-values representing heterogeneity were obtained by performing one degree of freedom trend tests, treating coffee consumption as a semi-continuous variable. As there exists prior evidence that certain tumour characteristics such as ER status are associated with age at diagnosis [22], and that coffee consumption is significantly associated with age at diagnosis [23], every model fitted in the case-only analysis was also adjusted for age at diagnosis in years (continuous).

The validation analysis based on the MARIE study population was performed using Proc LOGISTIC in SAS version 9.2 (SAS Institute, Cary, NC, USA). The variable on coffee consumption was categorized in the same way as in the Swedish study with women who consumed one cup or less of coffee per day as the reference group. Unconditional logistic regression models were used to estimate ORs and corresponding 95% confidence intervals. To test for trend, we treated the four categories of cups per day as a continuous scored variable in the model statement only.

All statistical computations for the Swedish study were performed using R version 2.8 [24]. All *P*-values presented are two-sided tests of statistical significance at the 5% level.

## Results

Table 1 describes the characteristics of study subjects in both the Swedish and MARIE study with respect to several breast cancer risk factors. Age at menarche was weakly but positively associated with the disease (*P *= 0.057 in Swedish samples and *P *= 0.0026 in MARIE samples), a result consistent with the literature [25]. Family history of breast cancer, age at menopause, parity, age of first birth, recent BMI, use of HRT, alcohol consumption, physical activity and highest education level attained were strongly significant for breast cancer risk with effects in a direction consistent with those estimated in other epidemiological studies. Smoking for more than one year or more than 100 cigarettes was not found to be associated with breast cancer risk in the Swedish study (*P *= 0.176).

**Table 1:** Descriptive characteristics of post-menopausal women

	Swedish study				MARIE study			
	
Characteristic	Breast cancer cases/controls	Cases	Controls	***P***^**a**^	Breast cancer cases/controls	Cases	Controls	***P***^**b**^
Age (matching factor, years)	2,818/3,111	63.4 ± 6.7	64.3 ± 6.5	-	2,651/5,395	63.2 ± 5.6	63.2 ± 5.5	-
Age at menarche (years)	2,558/2,832	13.5 ± 1.4	13.6 ± 1.4	0.0565	2,369/4,580	13.6 ± 1.6	13.7 ± 1.7	0.0026
Age at menopause (years)	2,803/3,093	50.4 ± 3.5	50 ± 3.9	< 0.0001	1,614/3,417	49.5 ± 4.7	49.0 ± 5.0	0.0014
Parity (No. of live births)	2,818/3,110	1.8 ± 1.2	2.1 ± 1.4	< 0.0001	2,651/5,395	1.6 ± 1.1	1.7 ± 1.2	< 0.0001
Age at first birth (years)	2,373/2,753	25.3 ± 4.9	24.6 ± 4.6	< 0.0001	2,185/4,537	24.7 ± 4.6	24.5 ± 4.4	0.1879
Body mass index (kg/m2)	2,803/3,065	25.8 ± 4.2	25.5 ± 4.2	0.0009	2,651/5,390	23.2 ± 3.2	23.3 ± 3.3	0.7134
Alcohol consumption (g/day)	2,537/2,400	2.5 ± 4.5	2.1 ± 3.9	0.0032	2,649/5,389	8.9 ± 18.2	8.4 ± 13.8	0.1731
History of breast cancer in first degree relative (Yes, %)	2,745/2,607	15.6	7.7	< 0.0001	2,512/5,096	18.6	12.6	< 0.0001
History of benign breast disease (Yes, %)	2,818/3,111	13.9	8.1	< 0.0001	2,647/5,379	41.2	34.9	< 0.0001
Use of hormone replacement therapy (Ever, %)	2,811/3,087	48.4	40.3	< 0.0001	2,645/5,375	68.5	60.7	< 0.0001
Smoked > 1 year or > 100 cigarettes (Yes, %)	2,817/3,109	42.5	44.3	0.6490	2,651/5,393	46.2	46.4	0.8602
Education, categorical*	2,800/2,646			0.0797	2,649/5,395			0.4634
- elementary school		45.4	46.7					
- junior secondary school		24.8	21.5		low	56.5	57.2	
- high school		15.2	13.8		medium	28.4	28.7	
- university		13.9	12.0		high	15.1	14.1	
Physical activity one year before recruitment, categorical	2,794/2,936			0.0055	2,625/5,350			0.0031
- none		17.8	15.8			37.3	33.5	
- < 1 h per week		15.8	14.9			23.9	23.9	
- 1 to 2 h per week		33.6	28.1			17.1	18.2	
- > 2 h per week		32.0	35.6			21.7	24.4	

^a^*P*-values based on Wald tests. All logistic regression models adjusted for age at enrolment in years, continuous.

^b^*P*-values based on Wald tests. All logistic regression models adjusted for age at enrolment in years, continuous, and study region.

*MARIE study: education categorized into three levels: low, medium high.

^$^MARIE study: sports activities after the age of 50.

Table 2 summarizes the relationships between coffee consumption and other breast cancer risk factors in controls. The variables found to be significantly associated with coffee consumption among controls were HRT (*P *= 0.008), smoking (*P *< 0.0001) and highest education level attained (0.041).

**Table 2:** Associations of coffee consumption and breast cancer risk factors in Swedish study (controls only)

Breast cancer risk factors	Daily coffee consumption				
		
	≤ 1 cup	2 to 3 cups	4 to 5 cups	> 5 cups	***P *trend**^**a**^
					
Age at menarche, continuous (y)					
Mean (SD)	13.5 (1.5)	13.6 (1.4)	13.6 (1.4)	13.6 (1.5)	0.506
Controls (n) in each coffee consumption category	246	1,054	776	326	
					
Age at menopause, continuous (y)					
Mean (SD)	49.9 (4.1)	50 (4.1)	50 (3.8)	50.2 (3.6)	0.331
Controls (n) in each coffee consumption category	268	1,164	840	360	
					
Body mass index, continuous (kg/m2)					
Mean (SD)	25.4 (5.1)	25.4 (4)	25.4 (4)	25.4 (4.7)	0.979
Controls (n) in each coffee consumption category	264	1,158	840	362	
					
Alcohol consumption (g/day)					
Mean (SD)	2.1 (4.2)	2.2 (4.0)	2 (3.7)	2 (4.3)	0.411
Controls (n) in each coffee consumption category	252	1,056	761	327	
					
Hormone replacement therapy					
Proportion of ever users	0.46	0.44	0.42	0.37	**0.008**
	269	1,160	843	361	
					
Family history of breast cancer					
Proportion with family history of breast cancer	0.11	0.10	0.09	0.08	0.281
Controls (n) in each coffee consumption category	266	1,154	830	353	
Smoked > 1 year or > 100 cigarettes (No/Yes)					
Proportion of Yes	0.37	0.37	0.47	0.57	**< 0.0001**
Controls (n) in each coffee consumption category	270	1,170	846	363	
					
Parity/Age at first birth, categorical					
Proportion of nulliparous	0.14	0.12	0.09	0.10	0.876
Proportion of parous with age of first birth < 25 y	0.42	0.47	0.49	0.50	
Proportion of parous with age of first birth ≥ 25 y and < 30 y	0.30	0.30	0.26	0.29	
Proportion of parous with age of first birth ≥ 30 y	0.14	0.11	0.15	0.10	
Controls (n) in each coffee consumption category	270	1,171	846	363	
					
Education, categorical					
Proportion of highest education level					
to elementary school	0.39	0.52	0.50	0.50	**0.041**
- junior secondary school	0.25	0.22	0.23	0.24	
- high school	0.19	0.14	0.14	0.15	
- university	0.18	0.12	0.12	0.11	
Controls (n) in each coffee consumption category	269	1,168	843	362	
					
Physical activity one year before recruitment, categorical					
- none	0.17	0.19	0.16	0.22	0.624
- < 1 h per week	0.18	0.17	0.15	0.16	
- 1 to 2 h per week	0.31	0.31	0.34	0.26	
- > 2 h per week	0.35	0.33	0.36	0.36	
Controls (n) in each coffee consumption category	267	1,165	842	362	

^a^To obtain *P*-value for trend for coffee consumption treated as a continuous variable, linear regression analysis was performed for continuous risk factor variables (age at menarche (years), age at menopause (years), body mass index (kg/m^2^) and alcohol consumption (g/day)), logistic regression analysis was performed for binary risk factor variables (hormone replacement therapy, family history of breast cancer and smoking), and proportional odds logistic regression was performed for categorical risk factor variables (parity/age at first birth, highest education level, and physical activity one year before enrolment).

SD, standard deviation; y, year(s).

Table 3 shows the multivariate-adjusted OR estimates and corresponding 95% CIs of postmenopausal breast cancer for coffee consumption, overall and stratified by breast cancer tumour subtype based on ER and PR status, for the Swedish dataset. Results were shown for the following models: adjusted by the matching factor, age at enrolment, in years (continuous) only, and potential confounders (HRT ever/never, ever smoked > 1 yr or > 100 cigarettes, and education (elementary school, junior secondary school, high school or university)) and average daily alcohol consumption (g/day). A modest decrease in overall breast cancer risk was observed for the models adjusted for age only (OR_> 5 cups/day: ≤ 1 cup/day_: 0.80 ((0.64, 0.99), *P *= 0.028). When the model was further adjusted for HRT, smoking, education and average daily alcohol consumption, the protective effect on overall breast cancer risk was no longer found to be statistically significant. In the stratified analyses, significant reductions in the risk of ER-negative and PR-negative subtypes were observed, with the strongest effect being seen in ER-negative subtypes, for all models examined (OR_> 5 cups/day: ≤ 1 cup/day for multivariate model_: 0.43 (0.25, 0.72), *P *= 0.0003).

**Table 3:** Results of multivariate analysis in Swedish study, overall and stratified by hormone receptor status

Type of breast cancer	Daily coffee consumption	Controls/Cases	**OR**^**a**^	95% CI		**OR**^**bc**^	95% CI	
All	≤ 1 cup	270/298	1.00	reference		1.00	reference	
	> 1 to 3 cups	1,171/,277	1.00	0.83	1.20	1.01	0.84	1.23
	> 3 to 5 cups	846/904	0.96	0.79	1.16	1.00	0.82	1.22
	> 5 cups	363/328	0.80	0.64	0.99	0.84	0.66	1.06
	*P *trend		**0.028**			0.127		
								
ER-positive	≤ 1 cup	270/161	1.00	reference		1.00	reference	
	> 1 to 3 cups	1,171/685	0.99	0.80	1.23	1.03	0.82	1.30
	> 3 to 5 cups	846/501	0.99	0.79	1.23	1.07	0.84	1.36
	> 5 cups	363/169	0.77	0.59	1.00	0.87	0.65	1.15
	*P *trend		0.065			0.471		
								
ER-negative	≤ 1 cup	270/48	1.00	reference		1.00	reference	
	> 1 to 3 cups	1,171/148	0.74	0.52	1.05	0.77	0.53	1.11
	> 3 to 5 cups	846/92	0.58	0.40	0.85	0.60	0.40	0.89
	> 5 cups	363/31	0.44	0.27	0.71	0.43	0.25	0.72
	*P *trend		**0.0002**			**0.0003**		
								
PR-positive	≤ 1 cup	270/135	1.00	reference		1.00	reference	
	> 1 to 3 cups	1,171/603	1.04	0.83	1.31	1.05	0.83	1.34
	> 3 to 5 cups	846/434	1.01	0.80	1.29	1.09	0.85	1.40
	> 5 cups	363/144	0.77	0.58	1.02	0.84	0.62	1.13
	*P *trend		0.055			0.360		
								
PR-negative	≤ 1 cup	270/66	1.00	reference		1.00	reference	
	> 1 to 3 cups	1,171/212	0.75	0.55	1.02	0.85	0.61	1.17
	> 3 to 5 cups	846/150	0.71	0.52	0.98	0.75	0.53	1.05
	> 5 cups	363/55	0.59	0.40	0.88	0.67	0.44	1.01
	*P *trend		**0.014**			**0.034**		

^a^Odds ratio (OR) and corresponding 95% confidence intervals (CI) adjusted for matching factor (age at enrolment in years, continuous).

^b^ORs and corresponding 95% CI adjusted for age at enrolment, potential confounders (hormone replacement therapy (HRT), ever/never, ever smoked > 1 y or > 100 cigarettes, and education (elementary school, junior secondary school, high school or university)) and average daily alcohol consumption (g/day).

Significant *P *values are in bold.

ER, estrogen receptor; PR, progesterone receptor.

We next tested for heterogeneity in the effects of coffee consumption on hormone receptor status (ER and PR) in the Swedish study. The breast cancer risk reduction associated with higher coffee consumption was significantly higher for ER-negative compared to ER-positive tumours (*P *heterogeneity (age-adjusted) = 0.004). The effect of coffee consumption was, however, not significantly different by PR status (*P *heterogeneity (age-adjusted) = 0.230). The fitted multinomial logistic regression model (five categories; cases by ER/PR status and controls) without parameter restrictions was not found to perform better than the model with coffee consumption effect, restricted to be independent of PR status (*P *= 0.877). On the other hand, the unrestricted model performed better than the model with coffee consumption effect restricted to be independent of ER status (*P *= 0.034).

Motivated by the trend test results in the ER-negative cancers, we performed a validation analysis using the MARIE study. Table 4 shows the corresponding multivariate-adjusted OR estimates and corresponding 95% CIs of postmenopausal breast cancer for coffee consumption, overall and stratified by breast cancer tumour subtype based on ER and PR status, for the validation performed using the MARIE study. A modest protective effect of the same scale, but not reaching statistical significance, was observed in the MARIE study (OR_> 5 cups/day: ≤ 1 cup/day_: 0.87 (0.71, 1.07), *P *trend = 0.173). Although the difference in overall effect sizes for different breast cancer subtypes were less impressive in the validation dataset, the strongest protective effect was similarly observed for the ER-negative subtype (OR_> 5 cups/day: ≤ 1 cup/day_: 0.67 (0.43, 1.05), *P *trend = 0.326), followed by the PR-negative subtype (OR_> 5 cups/day: ≤ 1 cup/day_: 0.70 (0.49, 1.00), *P *trend = 0.280). The ORs, corresponding 95% CI, and *P*-values for trend were not altered by the introduction of smoking, alcohol consumption and other lifestyle factors.

**Table 4:** Validation results in the German MARIE study

Type of breast cancer	Daily coffee consumption	Controls/cases	**OR**^**a **^**(95% CI)**	**OR**^**b **^**(95% CI)**
All	≤ 1 cup	2,148/1,086	1.00 reference	1.00 reference
	> 1 to 3 cups	2,136/1,050	0.97 (0.88 to 1.08)	0.97 (0.87 to 1.07)
	> 3 to 5 cups	748/358	0.94 (0.82 to 1.09)	0.95 (0.82 to 1.10)
	> 5 cups	363/157	0.85 (0.70 to 1.04)	0.87 (0.71 to 1.07)
	*P *trend		0.128	0.173
				
ER-positive	≤ 1 cup	2,148/854	1.00 reference	1.00 reference
	> 1 to 3 cups	2,136/822	0.96 (0.86 to 1.08)	0.95 (0.85 to 1.07)
	> 3 to 5 cups	748/279	0.93 (0.79 to 1.09)	0.94 (0.80 to 1.10)
	> 5 cups	363/129	0.90 (0.72 to 1.12)	0.92 (0.74 to 1.15)
	*P *trend		0.221	0.302
				
ER-negative	≤ 1 cup	2,148/214	1.00 reference	1.00 reference
	> 1 to 3 cups	2,136/212	1.00 (0.82 to 1.22)	1.02 (0.83 to 1.24)
	> 3 to 5 cups	748/76	1.04 (0.79 to 1.36)	1.04 (0.78 to 1.37)
	> 5 cups	363/25	0.67 (0.44 to 1.03)	0.67 (0.43 to 1.05)
	*P *trend		0.288	0.326
				
PR-positive	≤ 1 cup	2,148/737	1.00 reference	1.00 reference
	> 1 to 3 cups	2,136/686	0.93 (0.83 to 1.05)	0.92 (0.82 to 1.04)
	> 3 to 5 cups	748/239	0.93 (0.78 to 1.10)	0.93 (0.78 to 1.10)
	> 5 cups	363/115	0.93 (0.74 to 1.17)	0.96 (0.76 to 1.20)
	*P *trend		0.299	0.363
				
PR-negative	≤ 1 cup	2,148/332	1.00 reference	1.00 reference
	> 1 to 3 cups	2,136/348	1.06 (0.90 to 1.24)	1.06 (0.90 to 1.25)
	> 3 to 5 cups	748/116	1.01 (0.80 to 1.26)	1.02 (0.81 to 1.28)
	> 5 cups	363/39	0.68 (0.48 to 0.97)	0.70 (0.49 to 1.00)
	*P *trend		0.194	0.280

Multivariate-adjusted OR estimates and corresponding 95% CIs of postmenopausal breast cancer for coffee consumption in the German MARIE study, overall and stratified by breast cancer tumour subtype based on ER and PR status.

^a^Odds ratio (OR) and corresponding 95% confidence intervals (CI) adjusted for matching factors age at enrolment in years (continuous) and study region.

^b^OR and corresponding 95% CI adjusted for age at enrolment in years (continuous) and study region potential confounders (hormone replacement therapy (HRT, ever/never), ever smoked > 100 cigarettes, and education (low, medium, high) and average daily alcohol consumption (continuous, in g/day)).

ER, estrogen receptor; PR, progesterone receptor.

## Discussion

Our main finding was that coffee consumption was associated with a strong reduction in breast cancer risk for the ER-negative tumour subtype. This effect was independent of HRT, smoking, highest education level attained, and average daily alcohol consumption.

In the multivariate-adjusted Swedish study, women who drank more than five cups of coffee per day were 57% (*P *= 0.0003) and 33% (*P *= 0.034) less likely to get the ER-negative and PR-negative disease, respectively, than the reference group. The effects were also found to be independent of PR status. Motivated by the trend test results of the ER-negative breast cancer subgroup using the Swedish data, we attempted to validate the results in the independent MARIE study, conducted in Germany. Though not reaching statistical significance, the strongest protective effect from heavy coffee consumption was similarly observed for the ER-negative subtype (OR_> 5 cups/day:__≤1 cup/day_: 0.67 (0.43, 1.05), *P *= 0.326) in the validation study.

We believe that, collectively, the results from the two studies in this paper support a protective effect of high intakes of coffee against ER-negative breast cancer. The weaker associations found within the MARIE study may perhaps be attributed to other factors related to coffee drinking, such as brewing method, bean type, and caffeine content. For example, Nilsson *et al*. [26] found potentially relevant chemical differences between filtered and boiled coffee. While a statistically significant decreased risk of breast cancer was observed in women drinking boiled coffee, filtered coffee was not found to be associated with the risk of breast cancer. One possible explanation of the weaker association with breast cancer risk in the MARIE study may thus be due to the primarily use of filtered coffee in Germany, and boiled coffee in Scandinavia [27].

Several other studies have also examined the relationship between direct measurements of coffee consumption or related variables and risk of ER-positive and ER-negative breast cancers. Some have reported results that are in accordance with the findings in this study. For example, a study by Larsson *et al. *[28] observed non-significant trends of increased ER-positive breast cancer risk and decreased ER-negative breast cancer risk with increased coffee intake per day in an independent Swedish cohort. Ganmaa *et al. *[29] observed a general protective effect of caffeine intake on breast cancer risk for both ER subtypes in the Nurses' Health Study, but the effect was only found to be significant for ER-positive breast cancers. On the other hand, caffeine intake was significantly associated with a higher risk of ER-negative breast cancer in the Women's Health study [30]. Although the results may appear inconsistent, it could be because no direct comparisons may be made between the different coffee-related variables measured. For instance, caffeine is only one out of the many different compounds contained in coffee, and thus caffeine intake is perhaps not a valid substitute for measuring the total effects of coffee consumption.

We speculate that coffee might contain compounds that differentially affect breast cancer of different ER subtypes. For example, trigonelline, a phytoestrogen present in coffee extract, has been suggested to activate ER through an estrogen-independent mechanism [9]. This compound is biologically active and is capable of stimulating cell growth of an ER-positive cell line at low concentrations. In addition, coffee has been shown to significantly contribute to levels of plasma enterolactone [31], a different phytoestrogen reported to be associated with a significant decrease in ER-negative breast cancer risk [12]. The presence of such compounds that specifically aggravate the tumourigenesis of ER-positive breast cancer and attenuates the risk of ER-negative breast cancer corroborates our finding that coffee consumption decreases breast cancer risk overall (both ER-negative and ER-positive), but the protection is less evident for the ER-positive subtype.

A limitation of our study is that receptor status was available for only 65.4% of the Swedish population. We have compared the characteristics between breast cancer cases with and without ER status (Table S1 in Additional file 1), and found no significant difference between the two groups, with the exception of age at first birth (*P *= 0.0393) and highest education level attained (*P *= 0.0003). The coffee consumption variable and risk factor data of both studies were self-reported, and could thus be subjected to errors in measurement. However, correlations between data collection via food frequency questionnaires and weekly diet records are generally high [28,32]. Coffee intake also tends to be very consistent from day to day over longer periods, and may thus be better recalled, thus strengthening the present analysis. In addition, we had limited our analyses to coffee consumption of cases and age-matched controls to before breast cancer diagnosis of cases to avoid the potential bias due to a change in the dietary habits resulting from disease diagnosis. Another concern is the availability of different kinds of coffee on the market - caffeinated, decaf, instant and brewed, among others. According to the European Coffee Report 2008 [33], decaffeinated coffee makes up 8.25% of the total trade of roasted coffee, while consumption of decaffeinated coffee in Sweden is negligible: less than 1%. However, the analysis of the MARIE study was only limited to caffeinated coffee.

Strengths of our study include it being a population-based study, its large sample size and detailed information on relevant variables: coffee consumption, reproductive and hormonal risk factors, and tumour characteristics. We have also obtained supporting evidence in a large, well-described and independent population-based study.

## Conclusions

In conclusion, we found no evidence that coffee consumption increases the overall risk of postmenopausal breast cancer. However, a high daily intake of coffee was found to be associated with a significant decrease in ER-negative breast cancer among postmenopausal women. Future studies are needed to confirm the effects of coffee consumption in the light of breast cancer subtypes.

## Abbreviations

BMI: body mass index; BRCA1: breast cancer 1, early onset; BRCA2: breast cancer 2, early onset; CI: confidence interval; ER: estrogen receptor; HRT: hormone replacement therapy; ICD: International Classification of Diseases; MARIE: Mamma Carcinoma Risk factor Investigation; OR: odds ratio; PR: progesterone receptor.

## Competing interests

The authors declare that they have no competing interests.

## Authors' contributions

JLi, KH, KC, JLiu and PH designed the study. PS, DFJ and JCC headed the validation effort. JLi, PS and KH conducted the statistical analysis. JLi drafted the manuscript, with substantial contributions from all authors mentioned.

## Supplementary Material

Additional file 1 **Table S1**. Descriptive characteristics of post-menopausal women with information on hormone receptor status and without. Click here for file
