# Supplementary material for: Coffee consumption modifies risk of estrogen-receptor negative breast cancer
Source: Breast Cancer Res. 2011 May 14;13(3):R49. doi: 10.1186/bcr2879 (PMC3218935; doi:10.1186/bcr2879)
Supplement: Additional file 1 — Table S1. Descriptive characteristics of post-menopausal women with information on hormone receptor status and without. [file bcr2879-S1.DOC]

**Table S1. Descriptive characteristics of post-menopausal women with information on hormone receptor status and without.**

| *Characteristic* | *With ER/*  *Without ER* | *With ER* | *Without ER* | *P^a^* |
| --- | --- | --- | --- | --- |
| Age  (matching factor, years) | 1843/975 | 63.3±6.7 | 63.6±6.7 | 0.2157 |
|  |  |  |  |  |
| Age at menarche (years) | 1667/891 | 13.5±1.4 | 13.6±1.4 | 0.3013 |
|  |  |  |  |  |
| Age at menopause (years) | 1835/968 | 50.4±3.5 | 50.2±3.5 | 0.1410 |
|  |  |  |  |  |
| Parity (No. of live births) | 1843/975 | 1.8±1.2 | 1.8±1.2 | 0.603 |
|  |  |  |  |  |
| Age at first birth (years) | 1569/804 | 25.5±5.0 | 25.0±4.7 | **0.0393** |
|  |  |  |  |  |
| Recent body mass index (kg/m2) | 1832/971 | 25.8±4.1 | 25.9±4.2 | 0.62211 |
|  |  |  |  |  |
| Alcohol intake (g/day) | 1662/875 | 2.5±4.6 | 2.4±4.4 | 0.547 |
|  |  |  |  |  |
| History of breast cancer in first degree relative (Yes, %) | 1795/950 | 16.1 | 14.7 | 0.310 |
|  |  |  |  |  |
| Use of HRT (Ever, %) | 1839/972 | 49.3 | 46.8 | 0.204 |
|  |  |  |  |  |
| Smoked >1year or  >100 cigarettes (Yes, %) | 1843/974 | 43.6 | 45.5 | 0.306 |
|  |  |  |  |  |
| Education, categorical | 1830/970 |  |  | **0.0003** |
| - elementary school |  | 43.1 | 49.6 |  |
| - junior secondary school |  | 25.3 | 24 |  |
| - high school |  | 15.6 | 14.6 |  |
| - university |  | 15.3 | 11.3 |  |
|  |  |  |  |  |
| Physical activity one year before recruitment, categorical | 1828/966 |  |  | 0.297 |
| - none |  | 17.5 | 18.4 |  |
| - < 1h per week |  | 15.3 | 16.6 |  |
| - 1-2h per week |  | 33.9 | 32.9 |  |
| - >2h per week |  | 32.5 | 31.2 |  |
|  |  |  |  |  |

^a^P-values based on Wald tests. All logistic regression models adjusted for matching factor age at enrolment in years, continuous.

ER: estrogen receptor: PR: progesterone receptor; HRT: hormone replacement therapy
